# Serum type and concentration both affect the protein-corona composition of PLGA nanoparticles

**DOI:** 10.3762/bjnano.10.101

**Published:** 2019-05-06

**Authors:** Katrin Partikel, Robin Korte, Dennis Mulac, Hans-Ulrich Humpf, Klaus Langer

**Affiliations:** 1 Institute of Pharmaceutical Technology and Biopharmacy, University of Muenster, Corrensstraße 48, 48149 Muenster, Germany; 2 Institute of Food Chemistry, University of Muenster, Corrensstraße 45, 48149 Muenster, Germany

**Keywords:** human serum, nanoparticles, poly(lactide-*co*-glycolic acid), protein corona, proteomics

## Abstract

**Background:** When nanoparticles (NPs) are applied into a biological fluid, such as blood, proteins bind rapidly to their surface forming a so-called “protein corona”. These proteins are strongly attached to the NP surface and confers them a new biological identity that is crucial for the biological response in terms of body biodistribution, cellular uptake, and toxicity. The corona is dynamic in nature and it is well known that the composition varies in dependence of the physicochemical properties of the NPs. In the present study we investigated the protein corona that forms around poly(lactide-*co*-glycolide) (PLGA) NPs at different serum concentrations using two substantially different serum types, namely fetal bovine serum (FBS) and human serum. The corona was characterized by means of sodium dodecylsulfate polyacrylamide gel electrophoresis (SDS-PAGE), Bradford protein assay, zeta potential measurements, and liquid chromatography–mass spectrometry/mass spectrometry (LC–MS/MS). Additionally, the time-dependent cell interaction of PLGA NPs in the absence or presence of a preformed protein corona was assessed by in vitro incubation experiments with the human liver cancer cell line HepG2.

**Results:** Our data revealed that the physiological environment critically affects the protein adsorption on PLGA NPs with significant impact on the NP–cell interaction. Under comparable conditions the protein amount forming the protein corona depends on the serum type used and the serum concentration. On PLGA NPs incubated with either FBS or human serum a clear difference in qualitative corona protein composition was identified by SDS-PAGE and LC–MS/MS in combination with bioinformatic protein classification. In the case of human serum a considerable change in corona composition was observed leading to a concentration-dependent desorption of abundant proteins in conjunction with an adsorption of high-affinity proteins with lower abundance. Cell incubation experiments revealed that the respective corona composition showed significant influence on the resulting nanoparticle–cell interaction.

**Conclusion:** Controlling protein corona formation is still a challenging task and our data highlight the need for a rational future experimental design in order to enable a prediction of the corona formation on nanoparticle surfaces and, therefore, the resulting biodistribution in the body.

## Introduction

Nanoparticle (NP)-based drug carrier systems offer outstanding opportunities in the treatment of many serious diseases. The unique physicochemical properties and the ability to bind a library of ligands make them advantageous for targeted drug delivery while minimizing side effects [1]. Among the different materials used to synthesize NPs, the biodegradable polymer poly(DL-lactide-*co*-glycolide) (PLGA) has attracted consideration due to its minimal systemic toxicity, favorable degradation characteristics, and sustained release properties. Furthermore, approval by the US Food and Drug Administration and the European Medicines Agency turned PLGA into a promising candidate as carrier material for NPs in future clinical applications [2]. However, despite intensive preclinical and clinical research only a few NPs have made it to clinical trials or market maturity [2–3]. One possible reason is the limited understanding of the interaction occurring at the interface between NPs and the physiological surrounding [3]. Once in contact with biological fluids, such as blood, proteins adsorb onto the surface of NPs forming a protein corona [4]. Consequently, the synthetic identity of the NPs is replaced by a new biological identity that determines their physiological response including biodistribution, cellular uptake, trafficking, and toxicity [5]. Corona formation is a very dynamic process in nature, and it has been extensively investigated and comprehensively reviewed that the corona composition varies in dependence of the physicochemical properties of the NPs [5–6]. However, it is emerging that the characteristics of the biological environment, e.g., protein concentration [7–9], protein source [10–12], temperature [13], incubation time [14], and flow status [15], also play a determinant role in the formation of the protein corona. NPs can be administered via different routes, such as intravenous, intradermal, oral administration or via inhalation. During their journey through the body, NPs are exposed to changing biological microenvironments containing different protein compositions and concentrations affecting the corona formation with possible deep implications on the physiological response [3,16]. This emphasizes the great importance of examining the effects provoked by these environmental factors in order to successfully introduce and firmly establish new nanoparticulate dosage forms onto the market, thus offering further options to prevent and treat many major illnesses.

The main objective of the present study was to investigate the compositional evolution of the NP protein corona as a function of increasing serum concentration. Therefore, we produced NPs composed of the biodegradable polymer PLGA stabilized with poly(vinyl alcohol) (PVA) and subsequently incubated them with increasing amounts of either fetal bovine serum (FBS) or human serum to induce the formation of a protein corona. The use of two substantially different serum types further allowed us to assess the effect of the source origin on the protein adsorption. FBS is a common additive in standard cell culture media for many human cell lines and is frequently used as protein source in corona studies probably for economic reasons [14,17–18]. As human serum better mimics the in vivo conditions, we attempt to evaluate the difference of the protein source in order to contribute to a more rational design in future experimental studies.

Following corona formation and separation of the resulting NP–corona complexes from excess serum proteins we used sodium dodecylsulfate polyacrylamide gel electrophoresis (SDS-PAGE), zeta potential measurements, and liquid chromatography–mass spectrometry/mass spectrometry (LC–MS/MS) to study the composition of adsorbed proteins in detail. A quantitative analysis of corona proteins was conducted by Bradford assay after alkaline hydrolysis of PLGA–NPs. Finally, the consequences of corona formation on the interaction between NPs and cells were examined by in vitro incubation experiments with the human liver cancer cell line HepG2.

## Results and Discussion

### Compositional evolution of the protein corona with increasing serum concentration

Nanoparticles (NPs) of the present study are based on the biodegradable polymer PLGA stabilized with poly(vinyl alcohol) (PVA) and were prepared by an emulsion diffusion method [19]. Incorporation of Lumogen^®^ Red led to fluorescent labeled NPs easily trackable in cell culture experiments. Prior to NP incubation with increasing amounts of serum (FBS, human serum) and protein corona analysis the NPs were characterized accurately by PCS and zeta potential measurements. The obtained NPs showed a diameter of approximately 200 nm and a monodisperse size distribution with a PDI below 0.1 (see Table 1). The zeta potential of about −40 mV indicated colloidal stability due to electrostatic particle repulsion [14].

**Table 1:** Physicochemical characteristics of PLGA NPs (mean ± SD; *n* ≥ 3).

nanoparticle system	hydrodynamic diameter [nm]	polydispersity index	zeta potential [mV]	drug load [µg Lumogen^®^ Red/mg NPs]

PLGA NPs	214.6 ± 13.2	0.06 ± 0.02	−41.2 ± 8.1	—
PLGA NPs (Lumogen^®^ Red loaded)	221.0 ± 16.4	0.03 ± 0.02	−45.9 ± 1.5	8.14 ± 1.29

**Figure 1:**
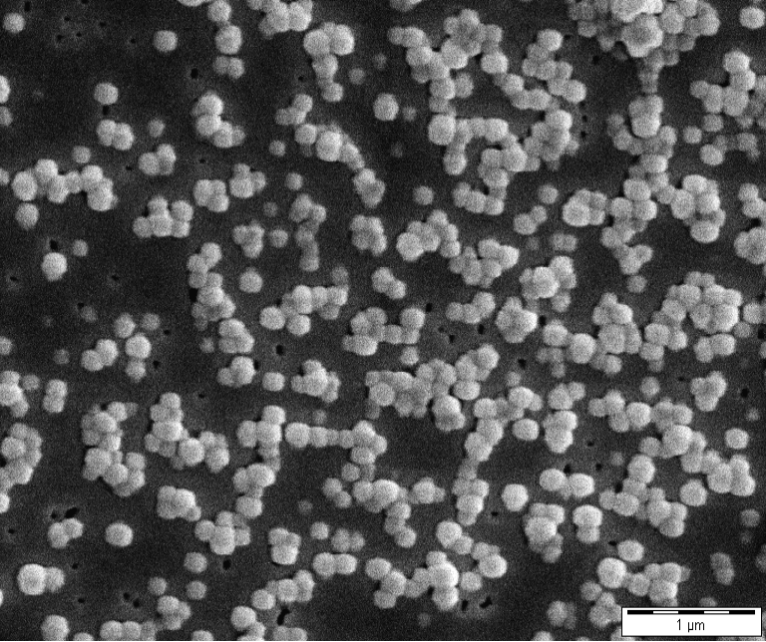
SEM confirmed the spherical shape of the PLGA NPs and the smoothness of the surface.

In our experimental setup we always referred to a constant surface area of NPs incubated with varying concentrations of serum. Surface asperities could lead to a higher surface area that would enhance the protein adsorption [20]. Therefore, we confirmed the spherical shape of the NPs and the smoothness of the surface by SEM (Figure 1). This allowed for a reliable examination of protein adsorption that will not be biased by effects of NP surface anomalies.

**Figure 2:**
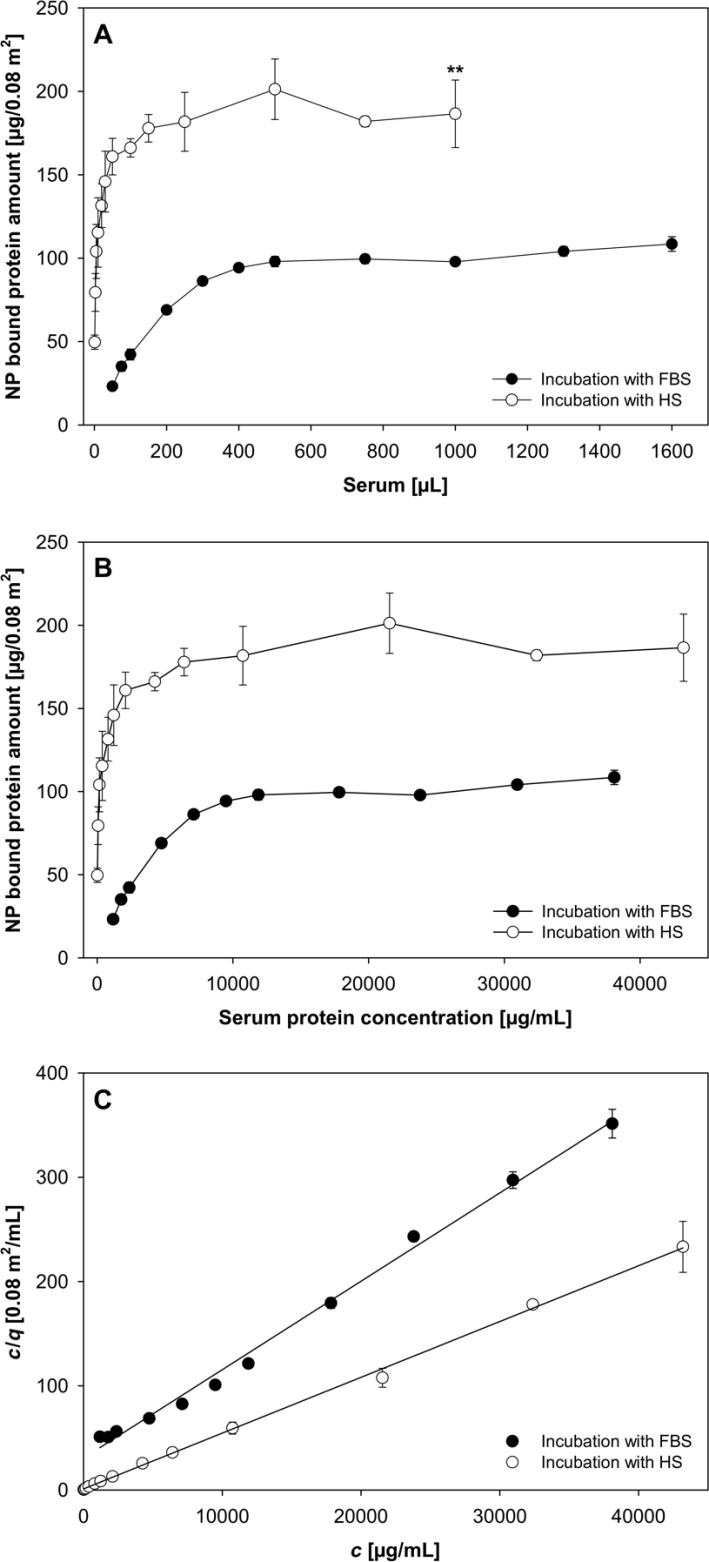
Adsorption of serum proteins on PLGA NPs (*n* ≥ 3; mean ± SD). (A) Quantification of the total amount of proteins adsorbed on NPs after incubation with different amounts of serum for 30 min at 37 °C and subsequent purification. (B) Langmuir adsorption isotherm. (C) Adsorption of FBS and human serum by PLGA NPs plotted according to Equation 1 using the data from (B). Abbreviations: fetal bovine serum (FBS), human serum (HS).

To focus on the evolution of the protein corona formed around PLGA NPs upon exposure to increasing amounts of serum, we applied the Bradford assay as a quantitative colorimetric approach to determine the total amount of proteins bound on PLGA NPs (Figure 2). NPs were incubated with either 50–1600 µL FBS or 1–1000 µL human serum for 30 min at 37 °C and subsequently purified in order to separate the NPs from unbound serum proteins. As can be seen from Figure 2A, the amount of NP-bound proteins at the lowest serum concentration level is 23.2 µg/0.08 m^2^ for FBS and 49.6 µg/0.08 m^2^ for human serum incubation. The amount of bound proteins steadily rises with increasing serum concentration until a plateau is reached. The plateau after incubation with human serum is twice as high as that after FBS incubation. The total amount of protein in serum prior to incubation was determined and was 47.7 mg/mL and 86.6 mg/mL for FBS and human serum, respectively. Afterwards, the equilibrium free fraction of protein in the incubation samples for each data point was calculated and the amount of protein adsorbed onto the surface of PLGA NPs was plotted as a function of protein that is free in solution (Figure 2B). The results exhibit the characteristic shape of a Langmuir adsorption isotherm for a multicomponent fluid indicating that equilibrium conditions could be achieved with increasing contents of serum in the incubation medium.

The Langmuir equation was arranged into the linear form and the adsorptive capacity (*q*_max_) of PLGA NPs for the different serum types was determined from the slope of *c*/*q* as a function of the concentration as shown in Figure 2C. The maximum amount of protein adsorbed after FBS incubation was 117.9 µg/0.08 m^2^ and for human serum incubation 186.9 µg/0.08 m^2^.

**Figure 3:**
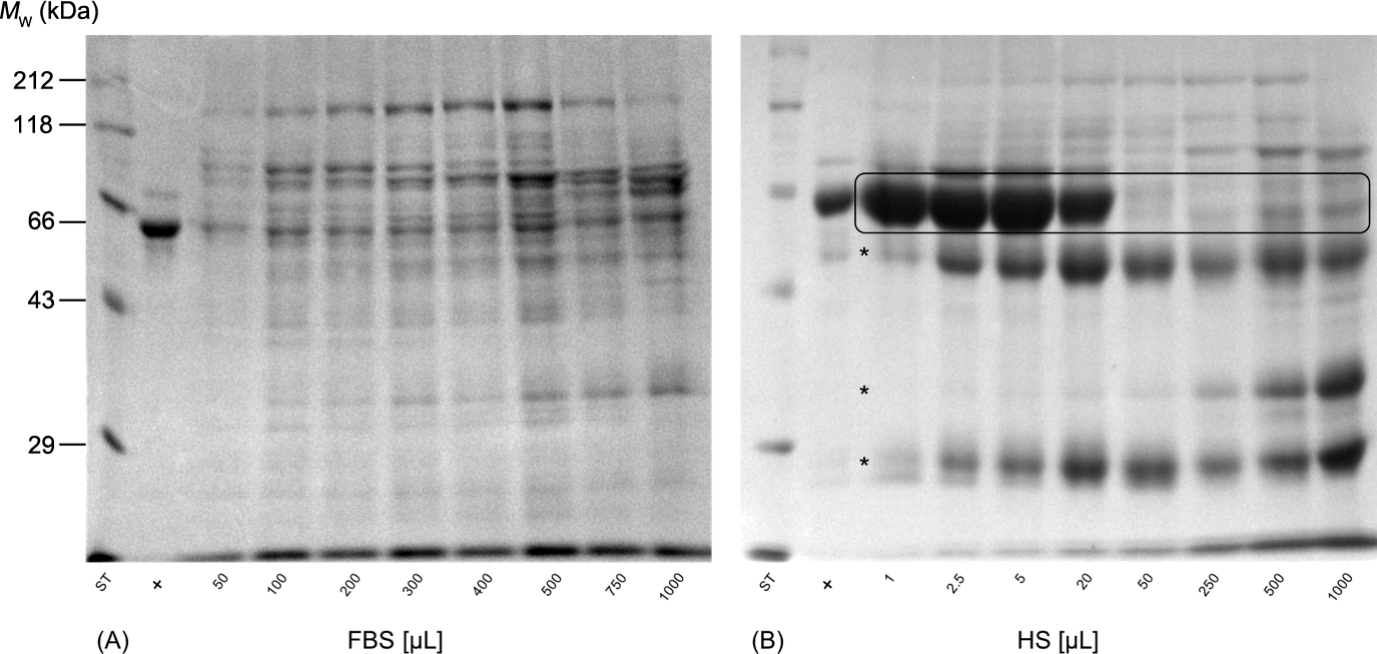
One-dimensional SDS-PAGE of adsorbed serum proteins obtained from the corona of PLGA NPs following incubation with increasing amounts of (A) FBS and (B) human serum. The molecular weights (*M*_W_) of the proteins in the marker lane on the left are reported for reference and positive controls (+) derived from pure serum diluted with purified water. Abbreviations: fetal bovine serum (FBS), human serum (HS).

In order to visualize the corona proteins after exposure of the NPs to increasing serum concentrations and to get more detailed information about the molecular composition, one-dimensional SDS-PAGE analysis was performed (Figure 3).

**Figure 4:**
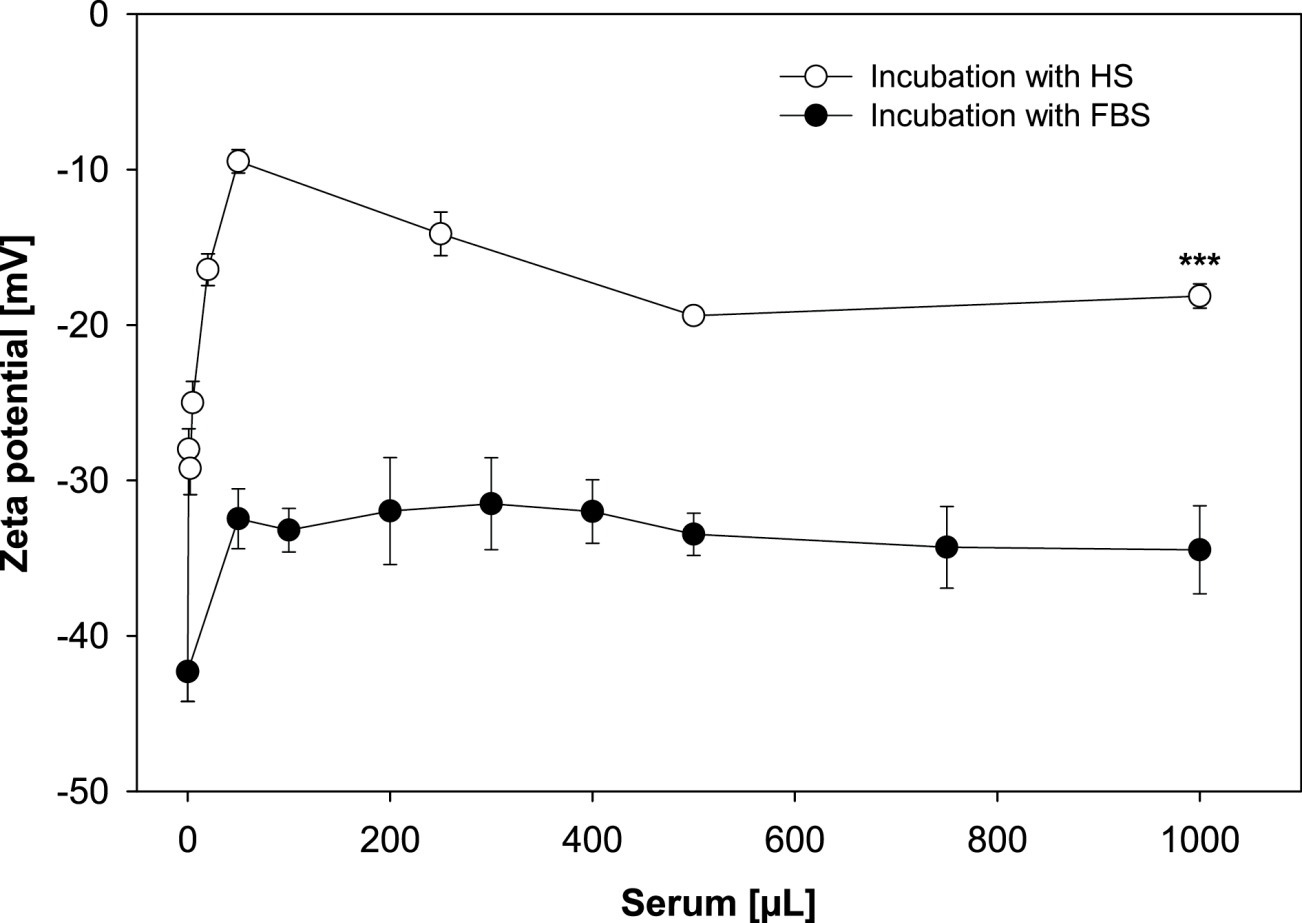
Surface charge evolution of PLGA NPs after exposure to different amounts of serum in the incubation solution. The differences in the mean values (mean ± SD; *n* ≥ 3) were statistically significant at the highest serum concentration indicating an enrichment of cationic proteins in the corona after exposure to human serum. Abbreviations: fetal bovine serum (FBS), human serum (HS).

The results illustrate a clear difference in corona protein identity and evolution trend for the two biological incubation fluids. The electrophoresis was carried out in triplicate. The protein pattern was reproducible and one gel for the incubation of PLGA NPs with increasing amounts of FBS is presented exemplarily in Figure 3A. The positive control is dominated by one major band corresponding to the molecular weight of serum albumin (67 kDa). In contrast, protein adsorption led to a highly selective enrichment of serum proteins on the surface of PLGA NPs [21], even at the lowest incubation concentration. The NPs are characterized by a protein pattern consisting of numerous protein bands ranging from 29 to 212 kDa. An accumulation of protein bands occurs between 43 and 118 kDa and a sharply defined band is located at the top of the gel above 118 kDa. Besides, three distinctive bands appear around 29 kDa. Figure 3A clearly shows that the composition of the hard corona remains stable over a wide range of FBS concentrations, while only the intensity of protein bands evolves until no further increase in staining intensity is visible. These findings reinforce the previously described assertion that the surface of PLGA NPs is more or less fully covered by proteins and a saturation effect occurred for FBS concentrations above a defined limit. Moreover, zeta potential measurements also provide evidence that the identity of corona proteins is quite stable when passing from low to high serum concentrations (Figure 4). In general, serum protein adsorption on negatively charged NPs leads to a decrease in zeta potential in dependence of amount and identity of the bound proteins [9,22]. The bare NPs display a zeta potential of −42.3 mV. Following incubation with 50 µL FBS it is significantly decreased to −32.5 mV suggesting that even for low serum concentrations a relatively complete protein layer was formed. With increasing amounts of FBS the zeta potential does not change considerably, which indicates that the qualitative composition of corona proteins remains constant. In order to address the effect of a higher ionic background the zeta potential of bare PLGA NPs as well as NPs incubated with 500 µL serum was measured in pure water and 0.2 mM NaCl, respectively. After water dilution PLGA NPs and NPs incubated with 500 µL FBS or human serum show zeta potentials of −43.2 mV, −34.0 mV and −21.4 mV, respectively. As could be expected the higher ionic background of 0.2 mM NaCl leads to a moderate reduction of the zeta potential to −33.0 mV, −30.5 mV, and −19.4 mV, respectively, but the graduation of the zeta potential values between the different samples remains the same. In brief, the incubation of PLGA NPs with increasing concentrations of FBS enabled us to modulate the amount of bound protein and to create a saturated surface while the identity of corona proteins was quite unchanged. These findings are consistent with former reports [7–9] in which Gräfe and collaborators revealed a saturation effect for the incubation of magnetic NPs at fetal calf serum concentrations above 75% (v/v) [8].

Nonetheless, the most intriguing observation of the present study is that the qualitative composition of the protein corona changed considerably with increasing human serum concentration in contrast to the incubation with FBS (Figure 3) even though the quantification of total amount of proteins bound to NPs reveals the same characteristic shape of an adsorption isotherm as for FBS incubation (Figure 2). Furthermore, it is noteworthy how selective and reproducible the adsorptive processes take place at the interface between particle surface and serum, considering that the serum is composed of more than 3700 different proteins [23].

**Figure 5:**
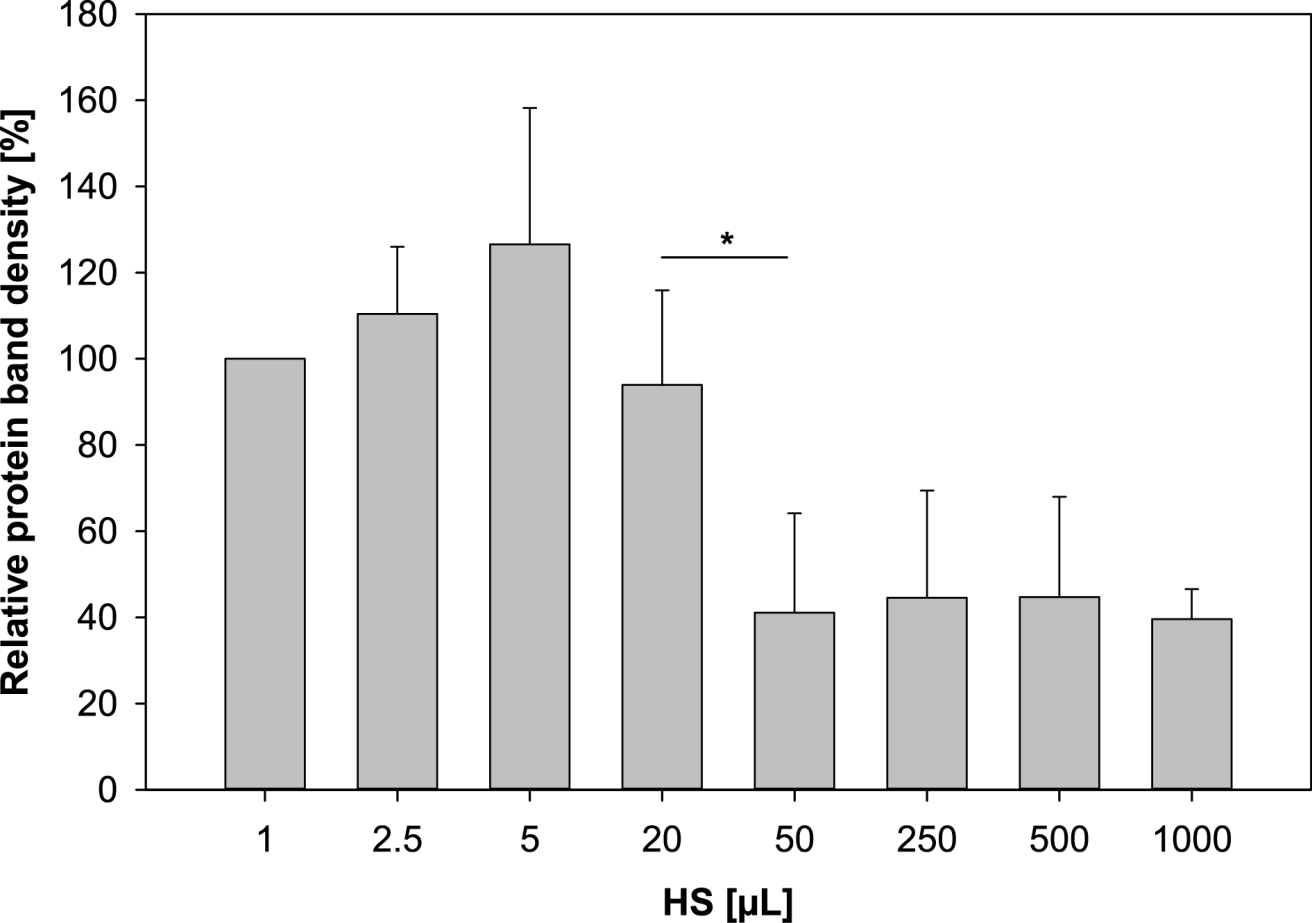
Semiquantitative densitometry analysis of the eight highlighted protein bands from Figure 3B (mean ± SD; *n* = 3). The first lane has been selected as standard and the density of the other bands is given relative to this selected band. Abbreviations: human serum (HS).

In the case of human serum the SDS-PAGE results (Figure 3B) illustrate that the protein signature is dominated by one intense protein band corresponding to a molecular weight of 66 kDa following incubation with 1 µL human serum. Below, three faintly visible bands emerged (marked with asterisks) of which the intensity increases with higher serum content during incubation. However, the most striking shift in corona composition occurs from 20 to 50 µL addition of human serum. The predominant protein band (66 kDa) vanishes almost entirely and the overall profile is more complex. This observation was confirmed by a semiquantitative densitometry analysis of the eight highlighted protein bands at 66 kDa (Figure 5). The first band was used as standard and then, the relative density was calculated by dividing the density of each band by the density of the standard band. As can be seen, the relative protein band density significantly drops from 94.0% to 41.1% when increasing the content of human serum in the incubation medium to 50 µL.

In addition, zeta potential measurements also affirmed the variation in corona composition with increasing human serum concentration (Figure 4). At low concentrations, the zeta potential decreases continuously until the minimum value of −9.5 mV is reached after the addition of 50 µL human serum. After that, the potential rises again slowly to a value of about −19 mV. However, a further increase in human serum concentration does not lead to further changes of the zeta potential. This is in good agreement with the results illustrated in Figure 2 and Figure 3B and demonstrates that the amount and the molecular composition of the protein corona formed around PLGA NPs after exposure to human serum evolves quite significantly at low concentration levels but becomes constant when passing to higher serum concentrations.

Corona formation is a very dynamic, competitive and time-dependent process. In the early stage, low-affinity proteins with high abundance in serum, for instance, human serum albumin (HSA), adsorb onto the surface and are immediately replaced by high-affinity proteins with lower abundance and slower kinetics, such as apolipoproteins and immunoglobulins [4,24]. According to the predominant protein band at *M*_W_ = 66 kDa, we suggest an enrichment of HSA in the corona of the PLGA NPs following incubation with 1–20 µL human serum (Figure 3B). At low incubation concentrations highly abundant proteins tend to attach strongly onto the NP surface and form the hard corona although they exhibit a low affinity. With increasing human serum concentration in the samples, the total content of high-affinity proteins in the incubation medium increases. This enables them to act as competitive binders and enhances the desorption of proteins with lower binding affinity [4,7,9]. Hence, the formation of the protein corona in dependence on the human serum concentration may have deep implications for the prediction of the biological response and the pharmacokinetic behavior of colloidal drug carrier systems in the body. For instance, the coating of polystyrene NPs with HSA enhances their circulating lifetime in blood and reduces hepatic uptake clearance after intravenous injection into rats [25]. In contrast, some high-affinity proteins, for example immunoglobulins, facilitate phagocytosis by cells of the mononuclear phagocyte system (MPS) [26]. Furthermore, one has to consider that the ratio between NP surface and protein concentration is closely related to the administration route and dose [27]. As a result, controlling the protein-corona formation is still a challenging task and our study emphasizes the need of a careful control in future experimental designs in order to ensure predictability of NP biodistribution.

### The characteristics of the protein corona depending on serum type

One major goal of the present study was to compare the protein corona of PLGA NPs that were exposed to 1000 µL of either FBS or human serum in order to explore the effect of the origin of the protein source on the amount, surface charge, and identity of the adsorbed protein layer. As already discussed above, 1000 µL serum in the incubation medium create a saturated NP surface for both biological fluids and a further increase in serum content does not lead to changes in the corona formation. Accordingly, this allowed us to reliably address the aforementioned research topic without considering the variabilities caused by phenomena depending on the serum concentration.

**Figure 6:**
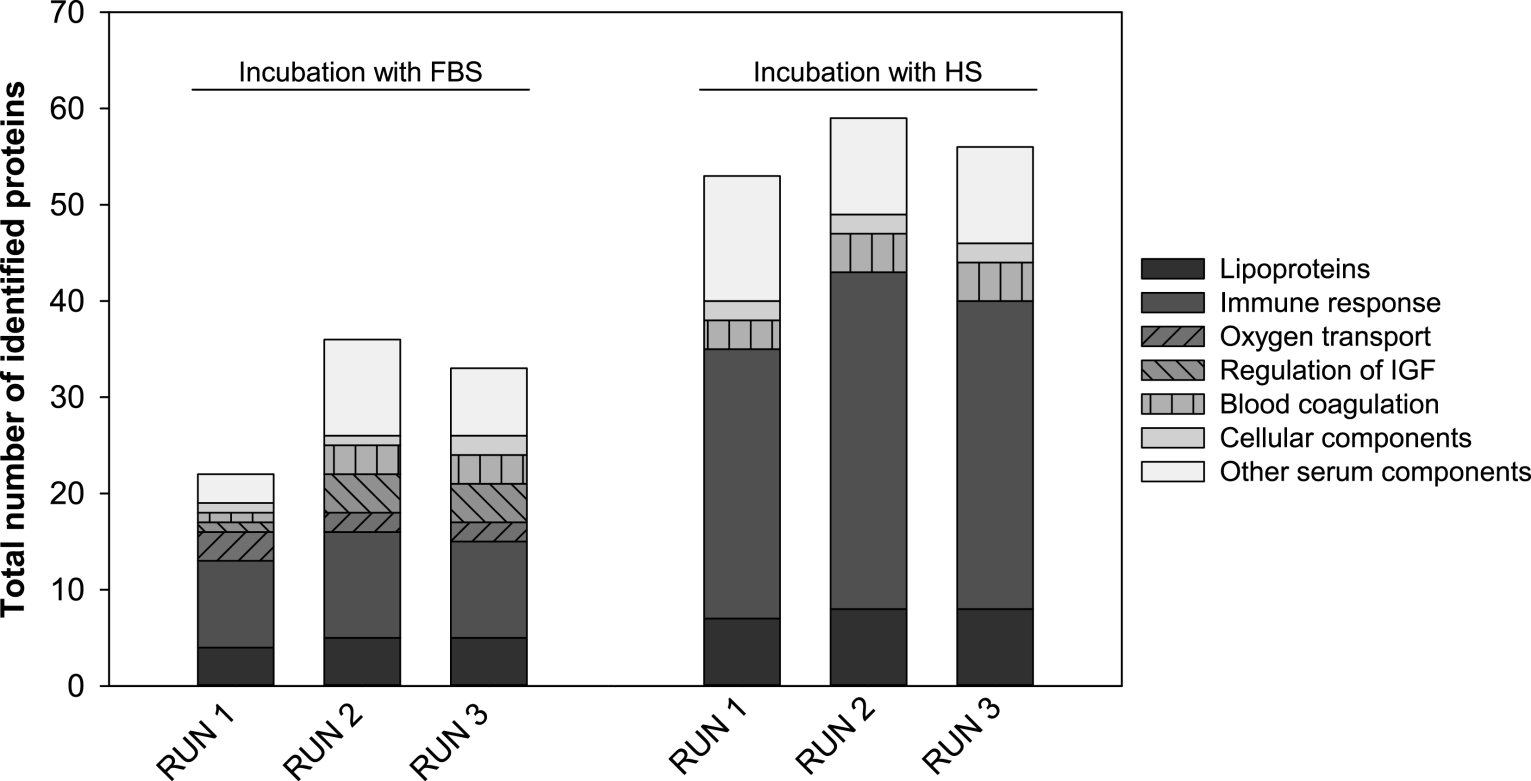
Bioinformatic classification of proteins identified in the corona of PLGA NPs after exposure to FBS or human serum. Proteins were analyzed by LC–MS/MS in three independent measurements (RUN 1–3) and grouped according to their function in blood. Abbreviations: fetal bovine serum (FBS), human serum (HS), insulin-like growth factor (IGF).

The quantification of corona proteins was performed by Bradford assay and revealed a significant higher protein content after incubation with human serum (Figure 2). Nevertheless, this result did not only reflect the higher total concentration of proteins in pure human serum, which was determined right before incubation and was about twice as high as that of FBS. For instance, the protein amount bound to NPs following the addition of 50 µL serum is 160.91 µg/0.08 m^2^ for human serum and 23.18 µg/0.08 m^2^ for FBS. This provides first evidence that the different adsorptive capacities of PLGA NPs for the two serum types were due to a higher affinity of several human proteins to the NP surface. Furthermore, the protein patterns on SDS-PAGE gels confirm differences in the qualitative corona composition, and the significant lower zeta potential values after exposure to human serum indicate a higher level of cationic proteins in the corona (Figure 3 and Figure 4) [28]. Additionally, the hard-corona proteins were identified by a shotgun proteomics-based approach. The adsorbed proteins were tryptically digested and the resulting peptides were analyzed by LC–MS/MS and subsequently bioinformatically interpreted. In three independent replicates, we detected numerous individual proteins on the PLGA NPs surface in dependence of the origin of the incubation solution. The complete list of identified proteins including their physiological function in blood, *M*_W_ values, as well as their isoelectric point (pI) is shown in Table S1 and Table S2 (Supporting Information File 1). A total number of 53 to 59 different proteins was detected in the corona after incubation with human serum. In contrast, the corona of PLGA NPs exposed to FBS was found to be less enriched with a total number of 22 to 36 identified proteins. Towards a better understanding of protein adsorption processes in dependence of the local biological environment and its consequences for the fate of the NPs in vivo, we divided the identified proteins roughly into seven groups according to their physiological function in the body (Figure 6). A substantial part of the coronas consist of apolipoproteins. They are important components of lipoproteins that facilitate the transport of cholesterol, triglycerides, and phospholipids between plasma and cells [29]. Due to their lipid-binding domains, they are even more attracted to NPs composed of hydrophobic core materials [30–31] resulting in a prolonged circulation time in blood [18]. Moreover, covalent attachment of apolipoprotein A–I and apolipoprotein E to the NP surface enables drug transport across the blood–brain barrier [32]. Here, both proteins were identified as constituents of the corona of PLGA NPs underlining the involvement in cellular transport mechanisms and biodistribution.

It is noteworthy that, in particular, the number of identified opsonins depends on the choice of protein source. This is important because the presence of opsonins on the NP surface could shorten their circulation lifetime and increase their uptake by immune cells [10,26]. In particular, the number of proteins that are involved in immune response is considerably increased in the corona formed during human serum incubation (Figure 6). In Table S2 (Supporting Information File 1) many immunoglobulin (Ig) fractions are listed that have been exclusively identified in the hard corona after human serum exposure, for instance IgG L chain, immunoglobulin kappa variable 3-20 and immunoglobulin J chain. This is readily conceivable since fetal serum does not contain antibodies [17]. IgG is a major effector molecule of the humoral immune response and takes part in the general process of opsonization for presentation to macrophages [26]. Besides, it activates the classical pathway of the complement system. Immediately after binding of IgG to foreign materials it forms a complex with complement component C1. We detected subcomponents of the proteases C1r and C1s in the HS corona, which are part of the C1 complex and ensure complement amplification (Table S2, Supporting Information File 1) [33]. In a current study, Chen et al. emphasized the role of the complement system in blood clearance mechanisms of nanospheres [34]. However, one has to keep in mind that this is probably not the sole mechanism of elimination. For instance, Hu and co-workers supposed that adsorption of platelet factor 4 also promotes rapid clearance from the bloodstream (Table S2, Supporting Information File 1) [35]. In contrast, we identified hemoglobin subunits exclusively in the corona derived from incubation with FBS (Figure 6 and Table S1, Supporting Information File 1). Systemically administered NPs can interact with circulating blood cells resulting in an erythrocyte aggregation that is in many cases accompanied by hemoglobin release. Hemoglobin adsorbs to NP surfaces and therefore facilitates phagocytosis by macrophages [36].

The LC–MS/MS results are consistent with the data presented in Figures 2–4, leading us to the assumption that the origin of the protein source plays a crucial role in defining the biological identity of nanocarriers. Therefore, the results obtained in animal models are not directly applicable to humans. For example, due to the higher number of opsonins in the corona after NP incubation with human serum, one may expect a reduced circulation time in human patients [11]. Nevertheless, FBS is still widely used as protein source for the investigation of the reaction at the interface between NPs and biomolecules [14,18]. Consequently, we propose to examine the protein corona formation in the respective medium of the desired species (e.g., murine or human) for a better prediction of the NP biodistribution in vivo.

### Protein corona alters nanoparticle-cell interaction

It is now clearly emerging that the primary defining element of NPs in biological media is their protein corona, which is the entity actually seen by target cells [7]. In the present study we convincingly demonstrated that the composition of the protein corona formed around PLGA NPs strongly depends on the concentration and the species origin of the incubation solution. In order to investigate the biological consequences of varying protein corona characteristics on the interaction of PLGA NPs and cells, the human liver cancer cell line HepG2 was used for in vitro incubation experiments.

For an easy tracking of NPs in cell culture experiments, the fluorescent dye Lumogen^®^ Red was incorporated into the hydrophobic particle matrix. Due to the lipophilic properties of the dye molecule an average of 8.14 µg Lumogen^®^ Red/mg NPs was entrapped, which corresponds to a high embedding efficiency of 81.4% of the initially used substance (Table 1). Furthermore, Raudszus and colleagues showed that only a very slight Lumogen^®^ Red amount of less than 0.1% was released when polymeric NPs were incubated in serum-containing medium [37]. Therefore, we assumed a stable entrapment of the model drug even in the presence of proteins. Consequently, the Lumogen^®^ Red fluorescence measured in cell culture experiments could be directly associated with the interaction between NPs and cells and is not distorted by effects of dye leakage.

**Figure 7:**
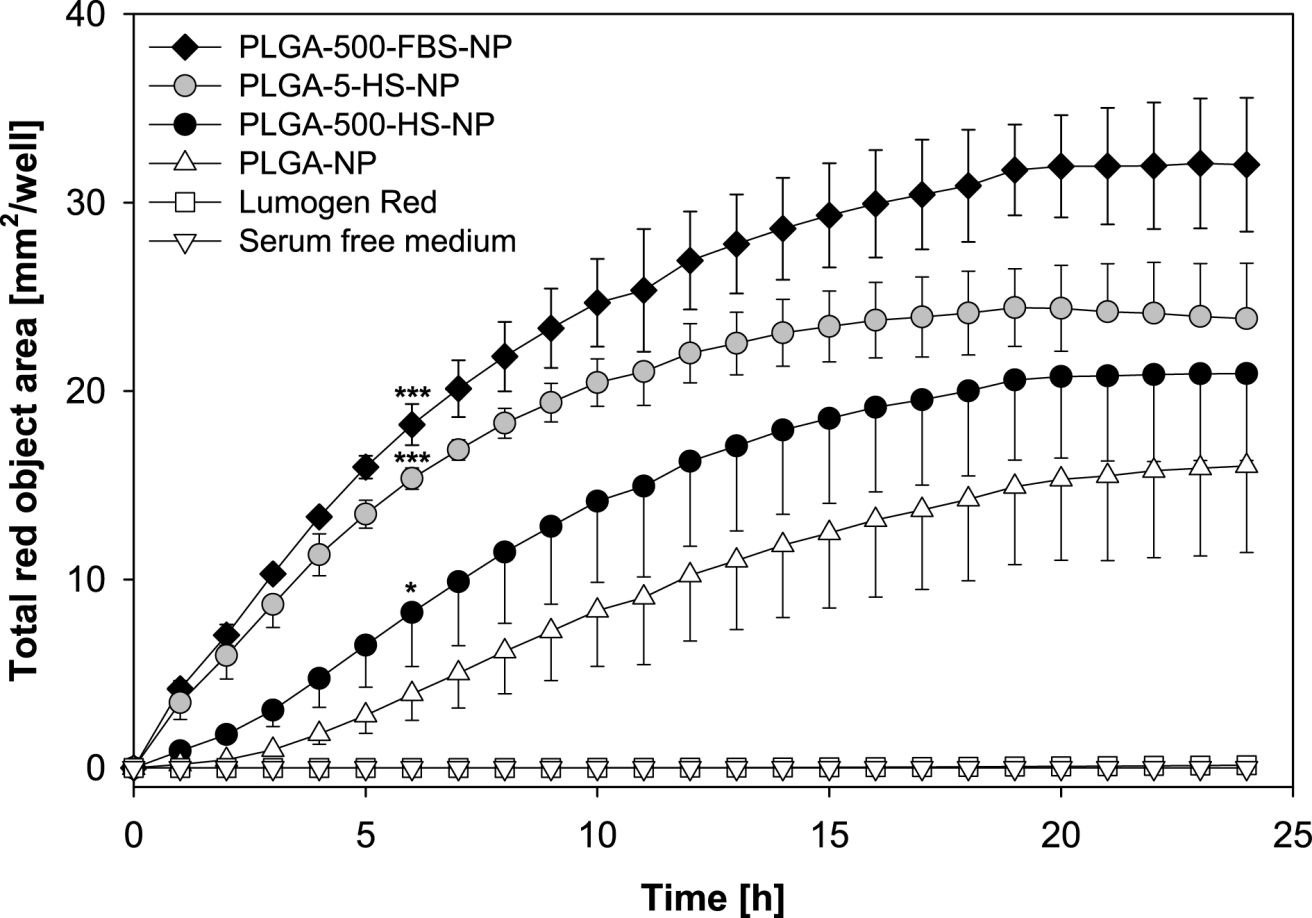
Time-dependent cell interaction of PLGA NPs with HepG2 cells in the absence or presence of a preformed protein corona (mean ± SD; *n* = 3). Statistical significances after 6 h versus PLGA NPs are marked with asterisks. Nanoparticles were loaded with Lumogen^®^ Red to visualize the nanoparticle-cell-interaction. Abbreviations: fetal bovine serum (FBS), human serum (HS).

The strategy used for this investigation was to preform coronas around PLGA NPs before exposure to cells, by incubating the NPs with 500 µL FBS (PLGA-500-FBS-NP), 500 µL human serum (PLGA-500-HS-NP) or 5 µL human serum (PLGA-5-HS-NP). Additionally, one sample was prepared by incubating the NPs with water instead of serum (PLGA NPs). We then added the NPs as well as the free dye Lumogen^®^ Red to the cells and monitored the cell interaction under serum-free conditions over a time period of 24 h by live-cell imaging (Figure 7). The results revealed that the interaction between the unformulated Lumogen^®^ Red and cells was neglectable. In contrast, all NP formulations showed an increasing cell interaction over time resulting in a plateau after approximately 18 h. The presence of proteins on the PLGA NP surface facilitates the adhesion to HepG2 cells compared to the bare particles. The significant lower zeta potential after exposure to serum indicated higher levels of cationic proteins in the corona (Figure 4) [28]. Therefore, we suppose a higher electrostatic attraction between the negatively charged cell membrane and the preincubated PLGA NPs [38]. Moreover, it became apparent that not only the mere presence of a protein corona but also the amount and type of protein adsorbed onto the surface determines the NP-cell interaction (Figure 7).

**Figure 8:**
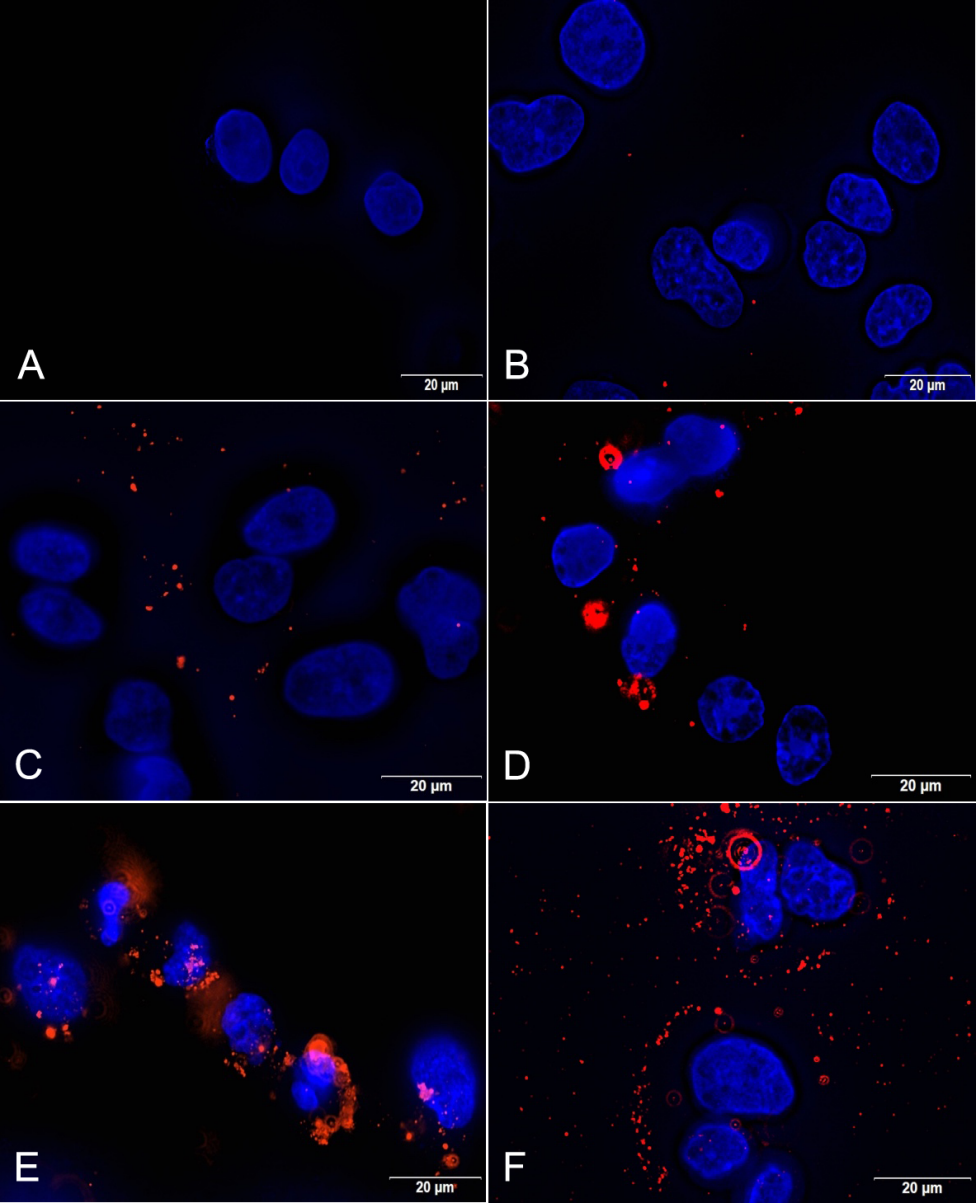
Visualization of the cell interaction of PLGA NPs after 6 h with HepG2 cells in the absence (C) or presence (D–F) of a preformed protein corona. Cell nuclei were stained with DAPI (blue) and Lumogen^®^ Red was detected through its autofluorescence (red). A: untreated cells; B: unformulated Lumogen^®^ Red; C: PLGA NPs; D: PLGA-500-HS-NP; E: PLGA-5-HS-NP; F: PLGA-500-FBS-NP. Abbreviations: fetal bovine serum (FBS), human serum (HS).

For instance, the interaction between cells and NPs after 6 h is about two- and about fourfold higher for PLGA-500-HS-NP and PLGA-5-HS-NP, respectively, when compared to the bare NPs. PLGA NPs preincubated with 500 µL FBS lead to the highest total red object area of 18.22 mm^2^/well. The results are consistent with the fluorescence microscopy images which were taken after an incubation time of 6 h (Figure 8). Furthermore, the images clearly demonstrate that all NP formulations are strongly attached to the cell membranes because they could not be removed by repetitive washings steps during sample processing. As can be seen in Figure 8F, lots of red dots are precisely located in the cell membrane indicating that treatment with PLGA NPs leads to an enrichment in the membrane. This step is usually considered as a prerequisite for a successive internalization by cells [38]. Besides, some of the proteins in the corona could mediate the interaction with cells by the recognition of specific receptor binding sites localized on the cell surface and thus induce different cellular behavior [17,38].

In summary, even in the case of NPs with the same chemical and physical properties, it is difficult to assess the biological response as long as the reaction at the nano–biointerface is not sufficiently understood. Therefore, the results of this study confirm the need to carefully evaluate the data acquired so far from in vitro studies in order to develop safe biomedical applications.

## Conclusion

We investigated the importance of selecting a proper physiological medium used for in vitro protein corona analysis. Therefore, we employed several analytical approaches to examine the protein corona that forms around PLGA NPs at different serum concentrations using two substantially different serum types. Our results showed that the amount of proteins bound to NPs increased when passing from low to high FBS concentrations while the identity of adsorbed proteins remained constant. In contrast, the corona composition of PLGA NPs incubated with human serum evolved considerably. Interestingly, the fraction of proteins displaying a *M*_W_ of about 66 kDa vanished almost entirely at higher concentrations. To further evaluate the effect of source origin on the corona formation we analyzed the proteins that bound under equilibrium conditions onto the NP surface in order to ensure comparability between the obtained results. Incubation with human serum led to a significantly higher amount of bound protein and the number of proteins involved in immune response was considerably increased indicating that circulation times in human patients may be different than that observed in animal models. However, one has to keep in mind that variations in analytical methods as well as measurement interpretation based on different databases can make direct comparisons of individual studies challenging.

Additionally, our data revealed that the characteristics of the protein corona altered the interaction between NPs and HepG2 cells underlining the importance of a careful control of experimental parameters in order to improve the interpretation and extrapolation of in vitro based studies. Consequently, our study represents a fundamental step towards establishing detailed relations between the interaction of PLGA NPs and the environmental surrounding, thus offering advances for accelerating the translation of new nanoparticulate dosage forms into the clinical practice.

## Experimental

### Reagents

The biodegradable polymer poly(DL-lactide-*co*-glycolide) (PLGA, Resomer^®^ RG 502 H), which was used as NP matrix, was obtained from Evonik Industries (Darmstadt, Germany). The steric NP stabilizer poly(vinyl alcohol) (PVA; Emprove^®^ exp 8-88, molecular weight approx. 67,000 g/mol, degree of hydrolysis 85–89%) was purchased from Merck KGaA (Darmstadt, Germany). The fluorescent dye Lumogen^®^ F Red 305 was kindly provided by BASF SE (Ludwigshafen, Germany). Fetal bovine serum (FBS) superior for NP incubation and cell cultivation was received from Biochrom AG (Berlin, Germany). Human serum was obtained from in.vent Diagnostica GmbH (Henningsdorf, Germany), donors informed consent documents and ethic votes are available. Roti-Load^®^1, Roti^®^-Mark STANDARD and all other chemicals used for sodium dodecyl sulfate polyacrylamide gel electrophoresis (SDS-PAGE) were delivered by Carl Roth GmbH & Co. KG (Karlsruhe, Germany). The dye Coomassie Brilliant Blue G-250 was purchased from VWR Life science AMRESCO (Solon, Ohio). Bovine serum albumin (BSA), DL-dithiothreitol (DTT), and iodoacetamide (IAA) were obtained from Sigma-Aldrich (Steinheim, Germany). Trypsin (sequencing grade) for protein digestion was obtained from Promega Corporation (Madison, USA). Urea was purchased from Acros Organics (New Jersey, USA).

Human liver cancer cells (HepG2) were kindly provided by the Institute of Food Chemistry of the University of Muenster, Germany. Phosphate-buffered saline (PBS), Dulbecco’s modified Eagle’s medium (DMEM) and all used supplements were received from Biochrom AG. Vectashield^®^ Mounting medium with DAPI was purchased from Vector Laboratories Inc. (Burlingname, USA) and wheat germ agglutinin (WGA) AlexaFluor^®^ 350 from Life Technologies (Carlsbad, USA). All other chemicals and organic solvents were delivered in the highest grade available.

### Nanoparticle preparation

The NPs were prepared by an previously described emulsification–diffusion method [19]. Briefly, 100 mg PLGA was dissolved in 2 mL ethyl acetate and subsequently added to 4 mL of an aqueous solution containing PVA (2%, w/w). The mixture was emulsified using a high-speed homogenizer (Ultra-Turrax^®^, S25NK-10G, IKA, Staufen, Germany) at 21,000 rpm for 30 min. The resulting pre-emulsion was poured into 6 mL of PVA solution (2%, w/w) and stirred overnight at room temperature to remove the organic phase. Finally, the NPs were purified by three steps of centrifugation (10 min, 16,000*g*) and following resuspension into ultrapure water. The NPs were referred to as PLGA NPs.

In order to visualize the NPs during cell culture experiments a fluorescent dye was embedded into the polymer matrix. Therefore, 100 mg PLGA and 1 mg Lumogen^®^ Red were dissolved in 2 mL ethyl acetate. All other preparation steps were conducted as described above.

### Nanoparticle diameter, size distribution and zeta potential

The hydrodynamic NP diameter and polydispersity index (PDI) were determined by photon correlation spectroscopy (PCS) using a Malvern Zetasizer Nano ZS system (Malvern Instruments Ltd., Malvern, United Kingdom). An appropriate volume of the different NP formulations was diluted in 2 mL ultrapure water in a disposable cuvette right before use and measured at a temperature of 22 °C using a backscattering angle of 173°.

The zeta potential was measured in the same instrument by laser Doppler microelectrophoresis to provide information about the surface charge of the NPs. The NP dilutions described above were transferred into a folded capillary cell and the determination was conducted at 22 °C.

### Morphological analysis of nanoparticles by SEM

A quantity of 3 µL diluted PLGA NP suspension (0.2 mg/mL) was applied on a 0.1 µm membrane filter (Isopore^TM^ membrane filter, Merck Millipore, Darmstadt, Germany) and dried overnight in a desiccator. Afterwards, the membrane filter was sputtered with gold (Sputter SCD 040, BALTEC, Liechtenstein) under argon atmosphere. SEM was performed on a CamScan CS4 microscope (Cambridge Scanning Company, Cambridge, United Kingdom) and the sample was visualized with an accelerating voltage of 10 kV, a working distance of 10 mm, and 20,000× magnification.

### Determination of Lumogen^®^ Red loading

The amount of embedded Lumogen^®^ Red was analyzed by HPLC using a fluorescence detector (Agilent Technologies 1200 Series, Agilent Technologies GmbH, Böblingen, Germany) [37]. Therefore, an aliquot of NP suspension corresponding to 1 mg of PLGA NPs was centrifuged for 30 min at 30,000*g*. Following this, the supernatant was discarded and the pellet was extracted with 1 mL acetonitrile for 2 h under slight shaking. Prior to HPLC analysis, the sample was centrifuged again (30 min, 30,000*g*) and 10.0 µL of the supernatant was injected onto a reversed-phase column (LiChroCart, Lichrosphere RP-18, 5 µm, 100 Å, 125 × 4 mm, Merck Millipore, Darmstadt, Germany). The elution was performed with pure acetonitrile as mobile phase at a constant flow rate of 1 mL/min and Lumogen^®^ Red was detected using a fluorescence detector at an excitation wavelength of 575 nm and an emission wavelength of 610 nm. Quantification was carried out using a calibration curve ranging from 0.1–10 µg/mL.

### Serum protein adsorption on nanoparticles

Serum protein adsorption on NPs was carried out according to a modified method described by Gossmann and co-workers [18]. Therefore, increasing amounts of either FBS (50–1600 µL) or human serum (1–1000 µL) were added to an aliquot of PLGA NP suspension corresponding to a total surface area (*A* = 4π*r*^2^) of 0.08 m^2^. Afterwards, samples were filled up to a total volume of 2 mL with ultrapure water and were incubated for 30 min at 37 °C under gentle shaking (1200 rpm). Finally, the samples were purified by at least two cycles of centrifugation (10 min, 16,000*g*) and redispersion into ultrapure water in order to remove unbound serum proteins. For the purpose of cell culture experiments, the Lumogen^®^ Red-loaded PLGA NPs were incubated likewise and resuspended after the last centrifugation step in DMEM supplemented with 1% (v/v) non-essential amino acids (NEA), 1% (v/v) L-alanyl-L-glutamine (200 mM), and 1% (v/v) penicillin/streptomycin (100 U/mL) (serum-free medium).

For the identification of corona proteins by LC–MS/MS analysis a larger particle surface area was required. Thus, 1000 µL of either FBS or human serum were added to an aliquot of PLGA NP suspension corresponding to a total surface area of 0.24 m^2^ and subsequently filled up to a total volume of 4 mL with ultrapure water. The further experimental procedure was conducted as described above.

### Quantification of total nanoparticle bound protein amount

For the quantification of the proteins in the corona, a photometric method based on a protein determination protocol by Bradford et al. [39] was used. The dye Coomassie Brilliant Blue G-250 binds to the proteins and causes a shift in the absorption maximum from 465 to 595 nm, which was monitored in a spectrophotometer Typ U-2900 (Hitachi High Technologies Corporation, Tokyo, Japan).

The previously obtained NP pellet was hydrolyzed with 100 µL NaOH 1 M and 400 µL purified water (15 min, 60 °C, 1200 rpm). Afterwards, 1.9 mL Bradford reagent was added to 100 µL of the hydrolyzed sample. Incubation for 10 min at 1200 rpm led to a stable protein–dye complex that was read at 595 nm. The amount of proteins bound to the NPs was quantified using a BSA calibration curve (0.05–0.5 mg/mL) with addition of 1 M NaOH.

In addition, the total protein amount in serum (FBS and human serum) was determined. Consequently, the free fraction of protein in serum for the different incubation conditions could be calculated by subtraction of the total amount of NP-bound proteins. This relation was represented as Langmuir adsorption isotherm for a multicomponent fluid applying the following equation:

[2] $$q=\frac{K_{\text{L}}\cdot q_{\max}\cdot c}{1+K_{\text{L}}\cdot c},$$

where *q* is the amount of solute (serum protein) adsorbed per weight of adsorbent (PLGA NPs), *c* is the serum protein concentration at equilibrium, *K*_L_ is a constant related to the enthalpy of adsorption and *q*_max_ is related to the surface area of the solid. This equation was arranged into the linear form

[1] $$\frac{c}{q}=\frac{c}{q_{\max}}+\frac{1}{K_{\text{L}}\cdot q_{\max}}.$$

The value of *q*_max_ is a measure of the adsorptive capacity of the adsorbent for the adsorbate under examination and was calculated for the adsorption of serum proteins on PLGA NPs.

### SDS-PAGE analysis of corona proteins

After protein adsorption and the last centrifugation step the pellet was resuspended under shaking (1200 rpm, 22 °C) in 30 µL reducing loading buffer (Roti-Load^®^1) overnight to desorb the proteins from the NP surface. Hereafter, the samples were centrifuged again (45 min, 30,000*g*) and the supernatant containing the proteins was transferred into a new reaction vessel and boiled for 5 min at 95 °C to denature the proteins. Subsequently, a 10% polyacrylamide gel was prepared and the samples as well as the protein standard (Roti^®^-Mark STANDARD) and serum positive controls were applied on the gel. For positive controls, serum was diluted 1:100 with ultrapure water. The SDS-PAGE was carried out at a constant voltage of 200 V for 1 h on an OmniPAGE mini system (Omnilab-Laborzentrum GmbH & Co. KG, Bremen, Germany). The resulting gel was fixed (79% water, 1% orthophosphoric acid, 20% methanol), stained with a colloidal Coomassie Brilliant Blue G-250 solution overnight and destained in methanol/water (1:3, v/v). Finally, Gel ix Imager (INTAS Science Imaging Instruments GmbH) was used for imaging.

In order to compare the density of protein bands after the visualization step a densitometric analysis was performed by using ImageJ software (Vers. 1.52s, https://imagej.nih.gov/ij/download.html). For data analysis, the density of the bands was expressed relative to the density of a selected standard band.

### Determination of zeta potential after serum protein adsorption

Following protein adsorption and the final centrifugation step the NP pellet was resuspended into 1 mL ultrapure water. 10 µL of the NP dispersion was diluted with 2 mL ultrapure water. Subsequently, the sample was transferred into a folded capillary cell and the zeta potential of the NP–protein complex was determined as described above.

### Identification of corona proteins by LC–MS/MS

Corona proteins were identified using a shotgun proteomics-based approach that has become the standard technique for the investigation of complex protein mixtures in recent years. Following tryptic digestion of the proteins, peptides were identified using LC–MS/MS on an orbitrap-based mass spectrometer and software-based data evaluation to interpret the peptide fragmentation data. In this study we referred to a protocol of Gossmann and co-workers [18].

### In-solution digestion of corona proteins

After washing and collecting the NPs with protein corona by centrifugation, the resulting pellet was resuspended in 100 µL TRIS buffer containing 6 M urea overnight at room temperature to desorb the proteins from the surface. Following this, the samples were centrifuged again (45 min, 30,000*g*) in order to isolate the desorbed proteins from the NPs. The supernatant consisting of corona proteins was mixed with 5 µL of 200 mM dithiothreitol for 1 h at room temperature to reduce the disulfide bonds. Subsequently, 20 µL of 200 mM iodoacetamide was added to the solution in order to alkylate cysteines. The reaction was conducted for 1 h in the dark. After that, the excess iodoacetamide was inactivated by another addition of dithiothreitol (20 µL, 200 mM) to the solution (1 h, 1200 rpm, 22 °C). To prepare tryptic in-solution digestion of proteins, the samples were diluted to a total volume of 1000 µL with ultrapure water to a final concentration of 0.6 M urea to maintain the activity of trypsin. Next, 10 µL of ice-cooled trypsin solution (200 ng/µL) was added to the diluted samples and digestion was carried out overnight under slight shaking (37 °C, 900 rpm). Finally, the reaction was stopped by adjusting the pH to below 6 with glacial acetic acid and the samples were filled up to a total volume of 2 mL with ultrapure water for the further experimental procedure.

### Sample preparation for mass spectrometry

To prepare for a successful mass spectrometric analysis of the peptides the samples were purified by solid-phase extraction in order to remove salts and undesired impurities. Briefly, Strata^TM^-X 33u RP 30 mg/1 mL columns (Phenomenex, Aschaffenburg, Germany) were sequentially activated and equilibrated with 1 mL methanol and 1 mL 1% formic acid before the digestion solutions were applied onto the columns in aliquots of 1 mL. Thereafter, the samples were desalted by washing with 1 mL of purified water. Then, the stationary phase including the peptides was rinsed with 600 µL of Eluent I (MeOH/H_2_O + 1% FA (5:5)) followed by 400 µL of Eluent II (MeOH/H_2_O + 1% FA (7:3)). The eluents were collected and evaporated nearly to dryness in a Thermomixer^®^ comfort (40 °C, 300 rpm) under nitrogen atmosphere. Finally, the residue was redissolved in a mixture of 100 µL acetonitrile, formic acid, and purified water (3:1:96, v/v) and the samples were stored at −20 °C until LC–MS/MS analysis.

### Mass spectrometric detection of peptides

Data were acquired on an LTQ Orbitrap XL hybrid ion trap-orbitrap mass spectrometer coupled to an Accela HPLC system (both Thermo Scientific, Dreieich, Germany). The injection volume was 20.0 µL and LC separation of enzymatic digests was carried out on an 2.1 × 150 mm, 2.6 µm Accurore C18 column (Thermo Scientific) at a constant flow rate of 250 µL/min employing the following gradient of ACN + 1% formic acid (A) and H_2_O + 1% formic acid (B): 3% A for 6 min, 3 to 12% A in 6 min, 12 to 35% A in 79 min, 35 to 60% A in 9 min, holding 60% A for 8 min, 60 to 3% A in 2 min and reequilibration at 3% A in 10 min. The mass spectrometer was operated in positive full scan and data-dependent mode (DDMS). Survey full-scan mass spectra (*m*/*z* 300–1500) were acquired in the Orbitrap (*r* = 30,000) and the two most intense ions were sequentially isolated, fragmented, and analyzed in the linear ion trap, using collision-induced dissociation (CID, normalized collision energy of 30% and an activation time of 30 ms). No charge states were rejected from fragmentation and target ions were dynamically excluded from repeated fragmentation for 45 s. Conditions for electrospray ionization (ESI) were: capillary temperature 225 °C; vaporizer temperature 350 °C; sheath gas flow 40 (arbitrary units); auxiliary gas flow 20 (arbitrary units); sweep gas flow 5 (arbitrary units); source voltage 3.5 kV; and tube lens 135 V.

### Data analysis for protein identification

For protein identification a database search was performed with PEAKS 7 (Bioinformatics Solutions, Waterloo, Canada) against the UniProt KB databases (*Bos taurus*, created 2016-04-25, 43803 entries; *Homo sapiens*, created 2016-03-12, 1073900 entries) using the PEAKS de novo algorithm and the enhanced target-decoy method (“decoy fusion”) for false discovery rate (FDR) estimation and result validation [40–41]. Search parameters were: (a) trypsin as specific enzyme, three missed cleavage allowed; (b) fixed modification: carbamidomethylation of cysteine and variable modification: oxidation of methionine, allowing for three variable PTM per peptide; (c) precursor mass error tolerance of 5 ppm; (d) fragment mass error tolerance of 1 Da. Proteins with a −log *P* value > 80 were considered to be reliable.

### Cell culture

HepG2 cells were cultivated in 75 cm^2^ flasks with DMEM supplemented with 10% (v/v) FBS, 1% (v/v) non-essential amino acids (NEA), 1% (v/v) L-alanyl-L-glutamine (200 mM), 1% (v/v) penicillin/streptomycin (100 U/mL) at 37 °C, 100% humidity in a 10% CO_2_ atmosphere. Cells were subcultivated twice a week at a ratio of approximately 1:5 after reaching 80–90% confluence or were used for cell culture experiments.

### Determination of nanoparticle–cell interaction by live-cell imaging

In order to investigate the interaction between PLGA NPs displaying a protein corona of different characteristics and HepG2 cells an IncuCyte^®^S3 Live-Cell Analysis Imaging System (Essen Bioscience, Inc., Michigan, USA) was used. Therefore, 1 × 10^5^ cells/well were seeded into a collagen coated 24-well plate and cultivated under serum-containing conditions as described above. After four days, the medium was replaced by 500 µL serum-free medium containing Lumogen^®^ Red-loaded PLGA NPs in a concentration corresponding to 0.2 nM Lumogen^®^ Red. Furthermore, unformulated Lumogen^®^ Red dissolved in serum-free medium with addition of 1% DMSO and serum-free medium without NPs were applied onto the cells as control. Subsequently, the cell interaction was monitored over a time period of one day taking nine images of each well every hour. Image channel red (excitation: 565–605 nm/emission: 625–705 nm) was used to determine Lumogen^®^ Red. Data evaluation was performed by calculation of the total red object area using the system software.

### Visualization of nanoparticle–cell interaction by fluorescence microscopy

For fluorescence microscopy, HepG2 cells were seeded at a density of 3 × 10^4^ cells/chamber on Millicell^®^ EZ slides (Merck KGaA, Darmstadt, Germany) and cultivated overnight. Afterwards, the serum-containing medium was removed and cells were incubated with Lumogen^®^ Red-loaded PLGA NPs, free Lumogen^®^ Red or serum-free medium as previously described. After 6 h, the cells were washed twice with phosphate-buffered saline (PBS^++^, containing Ca^2+^ and Mg^2+^) and fixed for 15 min with 4% paraformaldehyde at room temperature. After an additional washing step, the cells were covered with Vectashield^®^ Mounting medium with DAPI (Vector Laboratories Inc., Burlingname, USA) for nuclear staining.

All images were taken using a IX81 fluorescence microscope (Olympus, Hamburg, Germany) with filter systems including excitation at 360–370 nm, dichroic mirror at 400 nm, emission at 426–446 nm for DAPI and Alexa Fluor^®^ 350 and excitation at 535–555 nm, dichroic mirror at 565 nm, emission at 570–650 nm for Lumogen^®^ Red. All images were taken as multi-layer image stacks with a minimum of 15 images. To reduce out of focus fluorescence the stacks were processed by deconvolution (Wiener filter) using cellSens Dimensions Software 1.8.1.

### Statistical methods

All experiments were performed at least three times. The results are shown as average value with standard deviation. Significance tests were conducted with Sigma Plot 12.5 (Systat Software GmbH, Erkrath, Germany), using a one-way ANOVA test with the Holm–Sidak post test. Significance levels were depicted as * for *p* ≤ 0.05, ** for *p* ≤ 0.01, and *** for *p* ≤ 0.001.

## Supporting Information

File 1 Proteins identified on NP surfaces.
