# Supplementary material for: Serum type and concentration both affect the protein-corona composition of PLGA nanoparticles
Source: Beilstein J Nanotechnol. 2019 May 6;10:1002–15. doi: 10.3762/bjnano.10.101 (PMC6541368; doi:10.3762/bjnano.10.101)
Supplement: File 1 — Proteins identified on NP surfaces. [file Beilstein_J_Nanotechnol-10-1002-s001.pdf]

BEILSTEIN JOURNAL OF NANOTECHNOLOGY

Supporting Information

for

Serum type and concentration both affect the protein-corona composition of PLGA nanoparticles

Katrin Partikel, Robin Korte, Dennis Mulac, Hans-Ulrich Humpf and Klaus Langer

Beilstein J. Nanotechnol. **2019,** *10,* 1002–1015. doi:10.3762/bjnano.10.101

Proteins identified on NP surfaces

License and Terms: This is a supporting information file under the terms of the Creative Commons Attribution License (http://creativecommons.org/licenses/by/4.0). Please note that the reuse, redistribution and reproduction in particular requires that the authors and source are credited.
The license is subject to the *Beilstein Journal of Nanotechnology* terms and conditions: (https://www.beilstein-journals.org/bjnano)

Table S1: Identified proteins on the surface of PLGA NPs after incubation with FBS. Proteins were identified (✓) in three independent measurements by LC–MS/MS and subsequent data evaluation. Proteins were listed in alphabetical order. Molecular weight (M_W) and functions are taken from the web page https://www.uniprot.org/ and the isoelectric point (pI) was calculated by https://web.expasy.org/compute_pi/. Functions were categorized as blood coagulation (BC); regulation of insulin-like growth factor (IGF); immune response (IR); lipoproteins (LP); other serum components (OC); oxygen transport (OT).

Acession number	Protein	Function	M_W [Da]	pI	RUN 1	RUN 2	RUN 3
B0JYQ0	ALB protein	OC	69,294	5.95	✓	✓	✓
P34955	Alpha-1-antiproteinase	OC	46,104	6.05		✓	
V6F9A2	Apolipoprotein A-I preproprotein	LP	30,276	5.71	✓	✓	✓
P81644	Apolipoprotein A-II	LP	11,202	7.8	✓	✓	✓
P19034	Apolipoprotein C-II	LP	11,061	5.67		✓	✓
Q0ZCB4	Apolipoprotein E	LP	27,074	8.87	✓	✓	✓
P17690	Beta-2-glycoprotein 1	LP	38,252	8.53	✓	✓	✓
Q32L58	C-C motif chemokine	IR	10,337	8.43	✓	✓	✓
Q29RR9	C-C motif chemokine ligand 14	IR	10,457	8.98		✓	
F1N0I3	Coagulation factor V	BC	222,214	5.52		✓	✓
Q2TBQ1	Coagulation factor XIII B chain	BC	75,167	6.34		✓	✓
P01030	Complement C4	IR	101,908	6.15	✓	✓	✓
A0A0F6QNP7	Complement component 3	IR	187,181	6.46	✓	✓	✓
Q3T0A3	Complement factor D	IR	27,878	7.64	✓	✓	✓
Q28085	Complement factor H	IR	140,374	6.43	✓	✓	✓
F1MC45	Complement factor H precursor	IR	96,593	5.98		✓	
Q32PI4	Complement factor I	IR	68,933	8.07	✓	✓	✓
Q17QC8	Complement factor properdin	IR	50,750	8.32	✓	✓	✓

P01035	Cystatin-C	OC	16,265	9.23	✓	✓	✓
A6QPP9	C-X-C motif chemokine	IR	12,609	9.3	✓	✓	✓
F1MYN5	Fibulin-1	OC	77,486	4.94		✓	✓
F1N1I6	Gelsolin	CC	85,687	5.86	✓	✓	✓
P02081	Hemoglobin fetal subunit beta	OT	15,859	6.51	✓	✓	✓
P01966	Hemoglobin subunit alpha	OT	15,184	8.07	✓	✓	✓
P02070	Hemoglobin subunit beta	OT	15,954	7.02	✓		
Q2F6J3	Insulin-like growth factor 2 preproprotein	IGF	10,782	7.48		✓	✓
P13384	Insulin-like growth factor-binding protein 2	IGF	34,015	7.13	✓	✓	✓
Q05716	Insulin-like growth factor-binding protein 4	IGF	27,890	7.10		✓	✓
F1MUK3	Insulin-like growth factor-binding protein 6	IGF	24,953	8.73		✓	✓
Q95121	Pigment epithelium-derived factor	OC	46,229	6,57	✓	✓	✓
P00735	Prothrombin	BC	70,506	5.97	✓	✓	✓
P82943	Regakine-1	IR	10,281	8.8		✓	✓
P04815	Spleen trypsin inhibitor I	OC	10,843	9,00		✓	✓
Q2KIS7	Tetranectin	OC	22,144	5.47		✓	✓
Q28194	Thrombospondin-1	OC	25,015	7.94		✓	✓
E1BH06	Uncharacterized protein	IR	192,764	7.2	✓	✓	✓
F1MLW7	Uncharacterized protein	OC	24,397	7.53		✓	
Q3ZBS7	Vitronectin	CC	53,575	5.92			✓

Table S2: Identified proteins on the surface of PLGA NPs after incubation with human serum. Proteins were identified (✓) in three independent measurements by LC–MS/MS and subsequent data evaluation. Proteins were listed in alphabetical order. Molecular weight (M_W) and functions are taken from the web page https://www.uniprot.org/ and the isoelectric point (pI) was calculated by https://web.expasy.org/compute_pi/. Functions were categorized as blood coagulation (BC); regulation of insulin-like growth factor (IGF); immune response (IR); lipoproteins (LP); other serum components (OC); oxygen transport (OT).

**Acession number**	**Protein**	**Function**	M_W **[Da]**	**pI**	**RUN 1**	**RUN 2**	**RUN 3**
G3GAU4	Anti-H1N1 influenza HA kappa chain variable region	IR	11,606	8.07		✓	✓
A2KBC5	Anti-HCS scFv	OC	25,223	8.29	✓		
Q96SB0	Anti-streptococcal/anti-myosin immunoglobulin lambda light chain variable region	IR	11,594	6.32	✓	✓	
Q8TCE1	Antithrombin-III	BC	29,092	9.03	✓	✓	✓
P02647	Apolipoprotein A-I	LP	30,778	5.56	✓	✓	✓
P02652	Apolipoprotein A-II	LP	11,175	6.27	✓	✓	✓
P06727	Apolipoprotein A-IV	LP	45,399	5.28	✓	✓	✓
P02654	Apolipoprotein C-I	LP	9,332	8.01	✓	✓	✓
P02655	Apolipoprotein C-II	LP	11,284	4.64	✓	✓	✓
P02656	Apolipoprotein C-III	LP	10,852	5.23			✓
P02649	Apolipoprotein E	LP	36,154	5.65	✓	✓	✓
Q8TCZ8	Apolipoprotein E	LP	6,664	10.00		✓	
A2NI60	BRE	OC	11,938	4.36	✓	✓	
D9IWP9	Beta-2-glycoprotein I	LP	36,255	8.37	✓	✓	✓
F5H2D0	Complement C1r subcomponent	IR	76,614	5.63	✓	✓	✓
F8WCZ6	Complement C1s subcomponent	IR	57,519	5.31	✓	✓	✓
B4E216	Complement C3	IR	122,592	6.5	✓	✓	✓

A0A140TA32	Complement C4-A	IR	187,763	6.7	✓	✓	✓
P08603	Complement factor H	IR	139,096	6.21	✓	✓	✓
Q03591	Complement factor H-related protein 1	IR	37,651	7.39	✓	✓	✓
V9GYE7	Complement factor H-related protein 2	IR	28,912	6.44	✓		
A6XNE2	Complement factor D preproprotein	IR	27,780	6.82		✓	✓
V9HW34	Epididymis luminal protein 213	OC	25,646	6.15	✓	✓	✓
P06396	Gelsolin	CC	85,697	5.9	✓	✓	✓
Q6MZV7	Hepatitis B virus binding protein	OC	52,121	7.51	✓	✓	✓
Q6ZW64	Ig alpha-1 chain C region	IR	53,321	6.07	✓	✓	✓
A0A075B6N8	Ig gamma-3 chain C region	IR	41,335	8.23	✓	✓	✓
P01861	Ig gamma-4 Chain C region	IR	35,941	7.18	✓	✓	✓
Q6PJF2	IGK@ protein	IR	25,521	6.14	✓	✓	✓
Q6P5S8	IGK@ protein	IR	25,773	5.94		✓	✓
S6AWD3	IgG L chain	IR	19,942	9.1		✓	
S6AWF4	IgG L chain	IR	19,842	6.57	✓	✓	✓
S6BGD6	IgG L chain	IR	24,824	6.81	✓	✓	✓
S6BAN6	IgG L chain	IR	22,920	8.56	✓	✓	✓
S6B294	IgG L chain	IR	20,110	8.96		✓	
S6C4R6	IgG L chain	IR	20,315	6.22	✓	✓	✓
Q8N355	IGL@ protein	IR	24,793	5.93	✓	✓	✓
Q6GMW3	IGL@ protein	IR	24,654	5.94	✓	✓	✓
Q6GMW4	IGL@ protein	IR	24,855	6.29		✓	✓
Q6NS95	IGL@ protein	IR	25,148	6.19	✓		✓
P01871	Ig mu chain C region	IR	49,307	6.35		✓	✓
P01591	Immunoglobulin J chain	IR	18,099	5.09	✓		

P06309	Immunoglobulin kappa variable 2D-28	IR	12,957	5.61	✓		
A0A0B4J1T9	Immunoglobulin kappa variable 3-15	IR	12,496	5.14		✓	✓
P01621	Immunoglobulin kappa variable 3-20	IR	10,729	4.85	✓	✓	✓
P18136	Immunoglobulin kappa variable 3-20	IR	14,089	4.85	✓	✓	✓
P06314	Immunoglobulin kappa variable 4-1	IR	14,966	5.09		✓	
B9A064	Immunoglobulin lambda-like polypeptide 5	IR	23,063	9.08			✓
Q0KKI6	Immunoglobulin light chain	IR	24,030	8,29	✓	✓	
Q14624	Inter-alpha-trypsin inhibitor heavy chain H4	IR	103,357	6.51	✓	✓	✓
A2NUT2	Lambda-chain (AA-20 to 215)	IR	24,655	7.54		✓	✓
V9HWP0	Pentaxin	OC	25,387	6.1	✓		
P02775	Platelet basic protein	OC	13,894	9.04	✓	✓	✓
P02776	Platelet factor 4	BC	10,845	8.93	✓	✓	✓
P10720	Platelet factor 4 variant	BC	11,553	9.3		✓	✓
P27918	Properdin	IR	51,276	8.32	✓	✓	✓
A0A096LPE2	Protein SAA2-SAA4	IR	23,353	9.1	✓	✓	✓
P00734	Prothrombin	BC	70,037	5.63	✓	✓	✓
Q6MZU6	Putative uncharacterized protein DKFZp686C15213	OC	51,099	7.85	✓		
Q6N093	Putative uncharacterized protein DKFZp686I04196	OC	46,061	7.63	✓	✓	✓
Q6MZX7	Putative uncharacterized protein DKFZp686M24218	OC	52,421	7.89	✓	✓	✓
P02768	Serum albumin	OC	69,367	5.92	✓	✓	✓
B2R5G8	Serum amyloid A protein	IR	14,808	9.04	✓	✓	✓
E9PQD6	Serum amyloid A protein	IR	13,546	6.28		✓	✓
E9PHK0	Tetranectin	OC	17,794	4.96	✓	✓	✓

Q6GMX0	Uncharacterized protein	OC	25,807	8.17	✓	✓	✓
Q6N095	Uncharacterized protein	OC	52,360	8.84	✓		✓
Q8TCD0	Uncharacterized protein	OC	26,235	8.24		✓	✓
D9ZGG2	Vitronectin	CC	54,306	5.55	✓	✓	✓
